# LAPTM4B gene polymorphism augments the risk of cancer: Evidence from an updated meta‐analysis

**DOI:** 10.1111/jcmm.13896

**Published:** 2018-09-25

**Authors:** Mohammad Hashemi, Gholamreza Bahari, Farhad Tabasi, Jarosław Markowski, Andrzej Małecki, Saeid Ghavami, Marek J. Łos

**Affiliations:** ^1^ Department of Clinical Biochemistry School of Medicine Zahedan University of Medical Sciences Zahedan Iran; ^2^ Student Research Committee Zahedan University of Medical Sciences Zahedan Iran; ^3^ ENT Department School of Medicine Medical University of Silesia in Katowice Katowice Poland; ^4^ Faculty of Physiotherapy The Jerzy Kukuczka Academy of Physical Education in Katowice Katowice Poland; ^5^ Department of Human Anatomy and Cell Science Rady Faculty of Health Sciences Max Rady College of Medicine University of Manitoba Winnipeg MB Canada; ^6^ Department of Molecular Biology School of Pharmacy with the Division of Laboratory Medicine in Sosnowiec Medical University of Silesia in Katowice Katowice Poland; ^7^ Centre de biophysique moléculaire UPR4301 CNRS CS80054 Orleans France

## 1 INTRODUCTION AND BACKGROUND

Lysosome‐associated protein transmembrane‐4 beta (LAPTM4B) has two alleles named as LAPTM4B*1 and LAPTM4B*2 (GenBank No. AY219176 and AY219177). Allele *1 has a single copy of a 19‐bp sequence in the 5` untranslated region (5`UTR), but allele *2 contains tandem repeats of 19‐bp sequence.^1^ LAPTM4B gene is located on long chromosome 8 (8q22.1) and contains seven exons that encodes two isoforms of tetratransmembrane proteins, LAPTM4B‐24 and LAPTM4B‐35, with molecular weights of 25 kDa and 35 kDa respectively. The LAPTM4B‐35′s primary structure is formed by 317 amino acid residues, and LAPTM4B‐24 comprised 226 amino acids. LAPTM4B, an integral membrane protein, contains several lysosomal‐targeting motifs at the C terminus and colocalizes with late endosomal and lysosomal markers. LAPTM4B is a proto‐oncogene, which becomes up‐regulated in various cancers. Preceding studies have examined the possible link between LAPTM4B polymorphism and susceptibility to several cancers,^1^, ^2^, ^3^, ^4^, ^5^, ^6^, ^7^, ^8^, ^9^, ^10^, ^11^, ^12^, ^13^, ^14^, ^15^, ^16^, ^17^, ^18^, ^19^, ^20^, ^21^, ^22^, ^23^, ^24^, ^25^, ^26^ but the findings are still inconsistent. Hence, the present meta‐analysis was designed to investigate the impact of LAPTM4B polymorphism on risk of cancer.

## 2 METHODS

A comprehensive search in Web of Science, PubMed, Scopus, and Google Scholar databases was done for all articles describing an association between LAPTM4B polymorphism and cancer risk published up to April 2018. The search strategy was “cancer, carcinoma, tumor, neoplasms,” “LAPTM4B, Lysosome‐associated protein transmembrane‐4,” and “polymorphism, mutation, variant.” Relevant studies included the meta‐analysis if they met the following inclusion criteria: (a) Original case‐control studies that evaluated the LAPTM4B polymorphism and the risk of cancer; (b) studies provided sufficient information of the genotype frequencies of LAPTM4B polymorphism in both cases and controls. The exclusion criteria were: (a) conference abstract, case reports, reviews, duplication data; (b) insufficient genotype information provided.

Data extraction was done by two independently authors. From each study, the following data were collected: the first author's name, publication year, country, ethnicity of participants, cancer type, genotyping methods of *LAPTM4B* polymorphism, the sample size, and the genotype and allele frequencies of cases and controls (Table 1).

**Table 1:** Characteristics of all studies included in the meta‐analysis

Author	Year	Country	Ethnicity	Cancer type	Source of control	Genotyping method	Case/control	Cases					Controls					HWE
								*1/1	*1/2	*2/2	*I	*2	*1/1	*1/2	*2/2	*1	*2	
Chen	2016	China	Asian	Renal cell carcinoma	PB	PCR	180/347	83	80	17	246	114	198	131	18	527	167	0.538
Chen	2016	China	Asian	Bladder cancer	PB	PCR	91/347	38	41	12	117	65	198	131	18	527	167	0.538
Chen	2016	China	Asian	B‐cell lymphoma	PB	PCR	162/350	87	64	11	238	86	199	133	18	531	169	0.549
Cheng	2008	China	Asian	Colon cancer	HB	PCR	253/350	113	112	28	338	168	199	133	18	531	169	0.538
Cheng	2008	China	Asian	Rectal cancer	HB	PCR	237/350	126	101	10	353	121	199	133	18	531	169	0.539
Cheng	2008	China	Asian	Oesophageal cancer	HB	PCR	211/350	123	80	8	326	96	199	133	18	531	169	0.539
Deng	2005	China	Asian	Lung cancer	PB	PCR	166/134	54	91	21	199	133	67	59	8	193	75	0.284
Ding	2018	China	Asian	B‐cell lymphoma	HB	PCR	162/350	87	64	11	238	86	199	133	18	531	169	0.538
Fan	2012	China	Asian	Breast cancer	HB	PCR	732/649	326	342	64	994	470	346	262	41	954	344	0.355
Hashemi	2014	Iran	Asian	Breast cancer	HB	PCR	311/225	137	163	11	437	185	104	117	4	325	125	0.009
Hashemi	2016	Iran	Asian	Prostate cancer	HB	PCR	168/176	102	55	11	259	77	79	87	10	245	107	0.025
Li	2006	China	Asian	Lung cancer	PB	PCR	131/104	70	56	5	196	66	57	36	11	150	58	0.155
Li	2012	China	Asian	Breast cancer	HB	PCR	208/211	90	100	18	280	136	129	76	6	334	88	0.185
Liu	2007	China	Asian	Gastric cancer	HB	PCR	214/350	88	107	19	283	145	199	133	18	531	169	0.483
Meng	2011	China	Asian	Cervical cancer	HB	PCR	317/413	127	153	37	407	227	225	163	28	613	219	0.775
Meng	2013	China	Asian	Endometrial cancer	HB	PCR	283/378	93	135	55	321	245	200	140	38	540	216	0.072
Meng	2017	China	Asian	Papillary thyroid carcinoma	HB	PCR	183/697	90	73	20	253	113	397	264	36	1058	336	0.352
Qi	2010	China	Asian	Liver cancer	HB	PCR	86/78	27	51	8	105	67	36	34	7	106	48	0.798
Shaker	2015	Egypt		Breast cancer	HB	PCR	88/80	36	40	12	112	64	45	29	6	119	41	0.661
Sun	2007	China	Asian	Lymphoma	HB	PCR	166/350	72	71	23	215	117	199	133	18	531	169	0.549
Sun	2008	China	Asian	Liver cancer	PB	PCR	190/175	72	110	8	254	126	99	67	9	265	85	0.586
Tang	2014	China	Asian	NSCLC	HB	PCR	392/437	158	171	63	487	297	226	176	35	628	246	0.928
Wang	2010	China	Asian	Pancreatic cancer	HB	PCR	58/156	24	26	8	74	42	74	67	15	215	97	0.976
Wang	2012	China	Asian	Liver cancer	HB	PCR	303/515	107	156	40	370	236	272	205	38	749	281	0.941
Wang	2013	China	Asian	Nasopharyngeal carcinoma	HB	PCR	134/327	74	48	12	196	72	163	145	19	471	183	0.69
Wang	2017	China	Asian	Pancreatic cancer	HB	PCR	233/842	98	116	19	312	154	435	350	57	1220	464	0.231
Xu	2012	China	Asian	Ovarian cancer	HB	PCR	282/365	122	115	45	359	205	231	108	26	570	160	0.009
Yang	2012	China	Asian	Gallbladder cancer	HB	PCR	91/155	34	45	12	113	69	88	57	10	233	77	0.850
Zhai	2012	China	Asian	Hepatocellular carcinoma	HB	PCR	102/135	37	52	13	126	78	—	—	—	205	65	—
Zhang	2014	China	Asian	Malignant melanoma	HB	PCR	220/617	101	102	17	304	136	336	246	35	918	316	0.248

Meta‐analysis was carried out using Revman 5.3 software (Copenhagen: The Cochrane Collaboration, 2014, The Nordic Cochrane Centre) and stata 14.1 software (Stata Corporation, College Station, TX, USA). For each study, Hardy‐Weinberg equilibrium (HWE) was determined by the chi‐squared test, in order to verify the representativeness of the study population.

The association between *LAPTM4B* polymorphism in relation to cancer risk was evaluated by pooled odds ratios (ORs) and their 95% confidence intervals (CIs). Pooled ORs and their 95% CIs for codominant, dominant, recessive, overdominant and the allelic comparison genetic inheritance models were calculated. The significance of the pooled OR was assessed by the *Z* test, and *P* < 0.05 was considered statistically significant. The choice of using fixed or random effects model was determined by the results of the between‐study heterogeneity test, which was measured using the *Q* test and *I* ^2^ statistic. If the test result was *I* ^2^ ≥ 50% or P_Q_ < 0.1, indicating the presence of heterogeneity, the random effect model was selected; otherwise, the fixed‐effects model was chosen.

The funnel plot was used to estimate the publication bias. The degree of asymmetry was measured using Egger's test; *P* < 0.05 was considered significant publication bias. To measure the potential influence of each study on the overall effect size, sensitivity analysis was performed.

## 3 RESULTS

**Figure 1:**
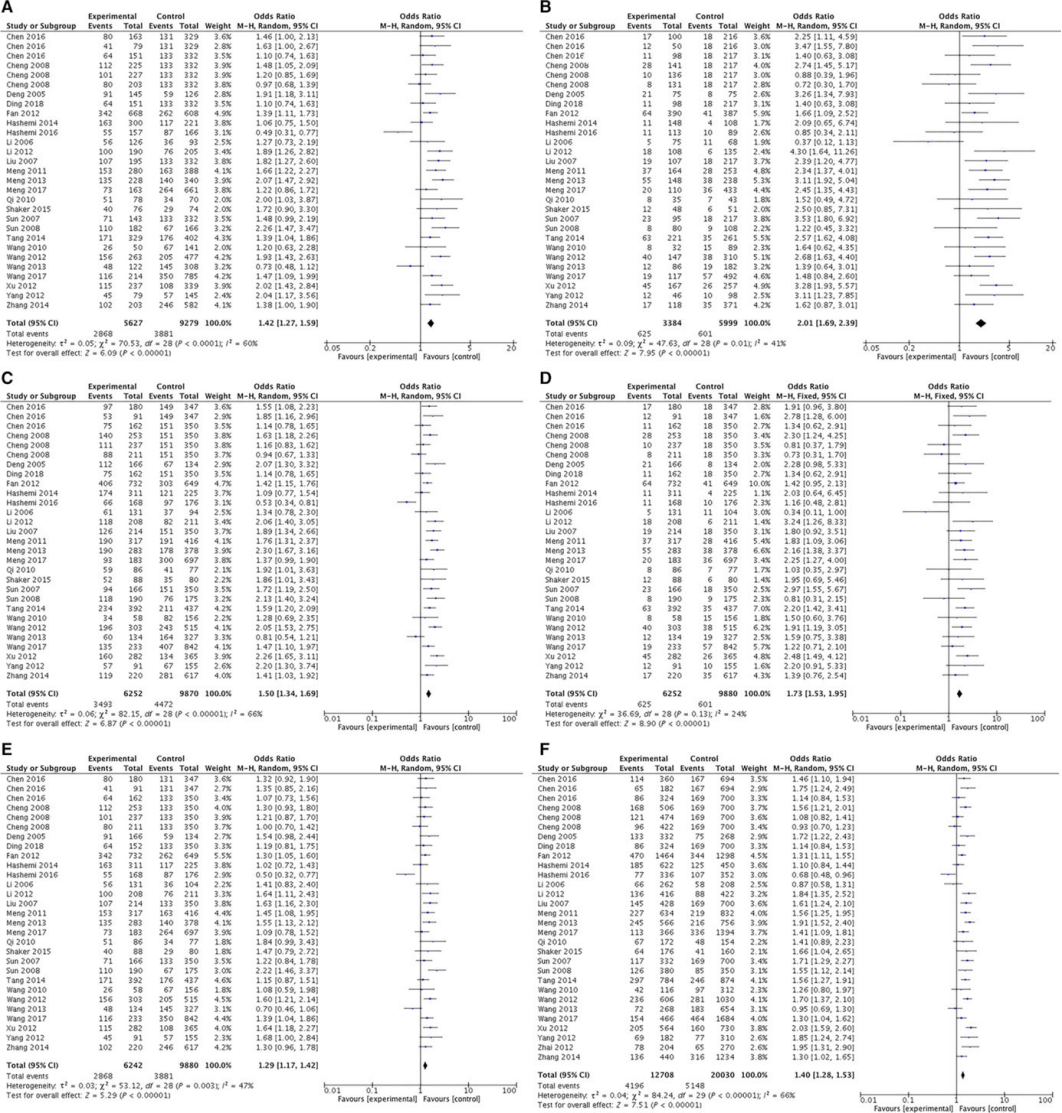
The pooled ORs and 95% CIs for the association between LAPTM4B polymorphism and cancer susceptibility. The forest plot for relationship between LAPTM4B polymorphism and cancer susceptibility for *2/2 vs *1/1 (A), *2/2 vs *1/1 (B), *1/2 + *2/2 vs *1/1 (C), *2/2 vs *1/2 + *1/1 (D), *1/2 vs *1/1 + *2/2 (E), and *2 vs *1 (F)

The characteristics and relevant data of the included studies are shown in Table 1. The results of the meta‐analysis revealed a significant association between *LAPTM4B* polymorphism and cancer susceptibility cancer in codominant (OR = 1.42, 95% CI = 1.27‐1.59, *P* < 0.00001, *1/2 vs *1/1; OR = 2.01, 95% CI = 1.69‐2.39, *P* < 0.00001, *2/2 vs *1/1), dominant (OR = 1.50, 95% CI = 1.34‐1.69, *P* < 0.00001, *1/2 + *2/2 vs *1/1), recessive (OR = 1.73, 95% CI = 1.53‐1.95, *P* < 0.00001, *2/2 vs *1/1 + *1/2), overdominant (OR = 1.28, 95% CI = 1.17‐1.41, *P* < 0.00001, *1/2 vs *1/1 + *2/2), and allele (OR = 1.40, 95% CI = 1.28‐1.53, *P* < 0.00001, *2 vs *1) inheritance model tested (Figure 1).

Stratifying according to cancer types proposed that LAPTM4B polymorphism significantly increased the risk of breast cancer, gastrointestinal cancer, gynaecological cancer, liver cancer, lung cancer, and lymphoma (data not shown).

The potential publication bias was evaluated using a Begg's funnel plot and Egger's test and the analysis suggested no publication bias for this meta‐analysis of the heterozygous codominant, dominant, recessive, overdominanat, and allele model (all *P*‐values for bias >0.05). We executed sensitivity analysis by neglecting a single study each time to reflect the influence of the individual data set to the pooled OR. The results indicated that the significance of pooled ORs for LAPTM4B polymorphism was not extremely influenced, suggesting the stability and reliability of the results in this meta‐analysis.

## 4 DISCUSSION

In the current study, we performed a meta‐analysis to find out the exact role of LAPTM4B polymorphism on risk of cancer. The results revealed that LAPTM4B polymorphism significantly increased the risk of cancer in codominant, dominant, overdominant, and allele genetic inheritance models. Stratification by cancer types suggested that LAPTM4B polymorphism is associated with the risk of breast cancer, gynaecological cancer, gastrointestinal cancer, liver cancer, lung cancer, and lymphoma. LAPTM4B is a proto‐oncogene that is overexpressed in various types of cancers. It has been proposed that overexpression of LAPTM4B‐35 promote proliferation, invasion, and migration. Its up‐regulation might be caused by gene amplification as well as transcriptional up‐regulation. LAPTM4B alleles have the same sequence except for one 19‐bp fragment for LAPTM4B *1 and two tight tandem fragments for LAPTM4B *2 in the 5′UTR of exon 1.^23^ The 19‐bp alteration in 5′UTR of the first exon of the LAPTM4B gene can shift the open reading frame (ORF), resulting in two alternate protein isoforms, LAPTM4B‐35 and LAPTM4B‐40. In conclusion, the finding of this meta‐analysis illustrated that LAPTM4B polymorphism may affect the risk of development of cancers.

## CONFLICT OF INTEREST

The authors declare no competing of interests.
